# Geographical and temporal variations in availability of national price negotiated novel anticancer drugs: a spatial statistical study based on two cross-sectional datasets in China

**DOI:** 10.3389/fphar.2025.1604008

**Published:** 2025-07-25

**Authors:** Ziqi Zhao, Shengwei Zhang, Tao Zheng, Ming Hu

**Affiliations:** West China School of Pharmacy, Sichuan University, Chengdu, Sichuan, China

**Keywords:** drug availability, health disparities and equity, GIS and spatial statistics, novel anticancer drugs, Retrospective databases

## Abstract

**Objective:**

The National Drug Price Negotiation (NDPN) has significantly reduced the prices and improved the nationwide availability of novel anticancer drugs (NADs) in China. However, geographical disparities in their availability remain concerning. This study aims to assess these spatial variations and temporal changes, and the determinants using geographic information system (GIS) and spatial statistical methods.

**Methods:**

Two cross-sectional datasets were used corresponding the implementation date of the 2023 NDPN list (1 January 2024) and 9 months after (1 October 2024). Data on drug-providing institutions were extracted from National Healthcare Security Administration (NHSA) platform. Drug availability was measured by the weighted supply number of drug-providing institutions per 1,000 cancer patients, analyzed separately for hospitals and retail pharmacies. Kernel density estimation (KDE) was used to visualize spatial distribution. The Theil index assessed inequality, and Moran’s index measured spatial clustering. Multiple linear regression (OLS) and geographically weighted regression (GWR) were employed to examine the influence of economic development and healthcare infrastructure on drug availability.

**Results:**

A total of 71 NADs in the 2023 NDPN list were analyzed. By October, drug-providing institutions had become more concentrated in the eastern coastal provinces compared to January. Availability improved in both hospitals and retail pharmacies, with higher levels observed in eastern and central provinces, with lower in the western provinces, especially in the Southwest. Inequality declined and spatial clustering increased for both hospital-based and overall availability across provinces (Theil index, hospital: 0.074–0.062, overall: 0.045–0.044; Moran’s I, hospital: 0.315–0.362, overall: 0.452–0.453). Both OLS and GWR models showed a significant and strengthening association between availability (in hospitals and overall) and GDP *per capita* [e.g., hospital: OLS coef, 0.787–0.833, p < 0.001; GWR mean coef (SD), 0.795 (0.047)−0.834 (0.044); overall: OLS coef, 0.744–0.794, p < 0.01; GWR mean coef (SD), 0.726 (0.119)−0.763 (0.161)]. Retail pharmacy-based availability was positively associated with the number of local chain pharmacies [OLS coef, 0.098–0.122, p < 0.05; GWR mean coef (SD), 0.084 (0.006)−0.107 (0.010)].

**Conclusion:**

The availability of price-negotiated NADs increasingly concentrated in economically developed and medically advanced eastern provinces, while remaining lower in southwest. Efforts should target economically underdeveloped areas.

## 1 Introduction

Cancer is a major public health challenge worldwide. According to estimates by the World Health Organization (WHO) in 2019, cancer was the first or second leading cause of death in 112 out of 183 countries and regions (Deo et al., 2022). China bears a substantial cancer burden, the ongoing population aging and the rise in unhealthy lifestyles are expected to further escalate the burden (Diao et al., 2025).

Modern cancer therapies often combine novel anticancer drugs (NADs) with conventional chemotherapy. NADs, including small molecule inhibitors, antibodies, and hormones, target and inhibit factors that provide survival advantages to tumor cells (Khader et al., 2022). In recent years, a significant number of NADs have been introduced worldwide. These drugs demonstrate clear clinical efficacy and can effectively improve adverse outcomes and prognosis. However, due to patent protection and technical monopolies, the prices of NADs are often relatively high (Xu, 2024). Aiming to curb soaring drug prices, the Chinese government introduced the National Drug Price Negotiation (NDPN) policy in 2017. The NDPN policy reduces the prices of innovative drugs by organizing regular price negotiations between the government and pharmaceutical companies. By 2024, six rounds of negotiations have been conducted. On 1 January 2024, the results of the 2023 NDPN were officially implemented through the National Reimbursement Drug List (NRDL). A total of 397 drugs were added to the reimbursement list through negotiations, including 71 NADs.

Although the prices have been reduced, the availability of NADs remains a significant concern. To enhance the availability of price-negotiated drugs, In 2021, the NHSA introduced the “dual-channel” management mechanism (National Healthcare Security Administration and National Health Commission, 2021), and provinces actively responded. This policy allowed retail pharmacies to sell these medications, which had previously been dispensed exclusively through hospitals in China. Today, retail pharmacies have become one of the primary channels for ensuring the supply of national price-negotiated drugs (Feng et al., 2025). However, the geographical variations in availability have once again raised concerns. Due to socio-economic factors and differences in regional healthcare infrastructure, not all areas have equal access to life-saving anticancer drugs (Gao et al., 2024).

Using spatial analytical methods to analyze the geographic distribution of drug-providing institutions is undoubtedly an effective strategy for studying the accessibility of medications--an integral part of the health system (Wirtz et al., 2017; Tharumia Jagadeesan and Wirtz, 2021). The density of drug-providing institutions per population within a region is also an important indicator for assessing whether availability can be achieved (Ward et al., 2014). The incorporation of geospatial information technologies and spatial statistical methods into health services research marks a shift from traditional non-spatial analyses, which have often overlooked the crucial principles of geography (Shi and Lin, 2020). Geographic Information Science (GIS) and spatial statistics have also paved the way for groundbreaking insights and advancements, particularly in investigating the nuanced aspects of spatial inequality in healthcare resource distribution (Dong et al., 2023) and in elucidating complex health-related issues (Song et al., 2024).

However, there has been no study to date using GIS and spatial statistical methods to evaluate drug availability in China. Moreover, current studies on drug availability often fall short in considering both hospitals and retail pharmacies. Therefore, this study aims to evaluate the availability of price-negotiated NADs across different types of drug-providing institutions and their geographical variations nationwide using methods such as kernel density estimation (KDE), The Theil index, Moran’s Index, and geographically weighted regression (GWR). Additionally, by analyzing two cross-sectional datasets, the study seeks to explore whether these geographical variations have shown a trend of widening after the NDPN policy.

## 2 Methods

### 2.1 Selection of medicines

In this study, we defined “national price-negotiated novel anticancer drugs” by referring to previous reviews of cancer therapies (Urruticoechea et al., 2010; Padma, 2015; Khader et al., 2022) as well as the Guidelines for Clinical Application of Novel Anticancer Drugs (2023 Edition) issued by National Health Commission (National Health Commission of the People’s Republic of China, 2024). Specifically, national price-negotiated NADs were defined as drugs that met both of the following criteria: (1) listed as negotiated items in the 2023 NRDL; (2) indicated for the treatment of malignant tumors and belonging to one of the three major therapeutic classes commonly regarded as innovative treatments—small molecule inhibitors, antibodies, and hormone therapies. Information on each negotiated drug was verified through the DrugBank (https://go.drugbank.com/drugs) to ensure therapeutic classification. Based on this selection process, a total of 71 drugs were included in the analysis. The full list is provided in Supplementary Table S1.

### 2.2 Data resources

The data on drug-providing hospitals and retail pharmacies for national price-negotiated NADs used in this study were extracted from the official platform of the NHSA (https://wx.nhsa.gov.cn/#/pages/negotiatingDrugs/negotiatingDrugs). This publicly accessible platform is designed to assist patients in locating designated providers for drug acquisition. The dataset covers a total of 63,266 hospitals—including tertiary, secondary, and primary hospitals, as well as community health centers and rural township health centers—and 172,694 retail pharmacies across 31 provincial-level administrative regions in mainland China (excluding the Macao Special Administrative Region, the Hong Kong Special Administrative Region, and Taiwan Province). The platform enables users to search either by drug, to view all institutions that supply it along with their addresses, or by institution, to view the full list of national price-negotiated drugs provided. The database is continuously updated by the NHSA to reflect the most current drug availability status.

Two cross-sectional datasets were collected on 1 January 2024, and 1 October 2024. The selection of these two time points enables a meaningful comparison of changes in drug availability from the initial implementation phase to a mature and stabilized phase of the policy cycle. According to the timeline of the NDPN policy implementation in China, 1 January 2024 marks the official implementation date of the updated national price-negotiated drug list, when the negotiated drug prices and reimbursement policies formally took effect (National Healthcare Security Administration and Ministry of Human Resources and Social Security of People’s Republic of China, 2023). This reflects the initial state of drug availability before the policy rollout. 1 October 2024, nearly 1 year into the implementation cycle and just prior to the 2024 NDPN [held in late October 2024 (National Healthcare Security Administration, 2024a)], captures a more stable and mature phase of the drug availability landscape.

To calculate availability and study its influencing factors, we also collected data on the population, cancer incidence rate, reginal economic development (GDP *per capita*) and healthcare infrastructure (number of tertiary A-grade hospitals and chain pharmacies) for each provincial-level administrative divisions (hereinafter referred to as “provinces”). The population data comes from the National Bureau of Statistics’ Seventh National Census results (https://www.stats.gov.cn), the cancer incidence rate data comes from the literature (Han et al., 2024), the GDP *per capita* data comes from the National Bureau of Statistics query platform (https://www.stats.gov.cn), and the healthcare infrastructure data comes from the China Pharmaceutical Innovation and Research Development Association and the National Medical Products Administration’s annual drug supervision statistics.

### 2.3 Data preprocessing and outcome indicators

Before calculating availability, we mapped the distribution of hospitals and retail pharmacies providing price-negotiated NADs. First, we converted the detailed addresses of drug-providing institutions into WGS-84 latitude and longitude coordinates using the geocoding interface of Gaode Map API (https://lbs.amap.com) and Python 3.12.0. We then imported these coordinates into QGIS and used KDE to calculate and plot the concentration of drug-providing institutions. KDE is a widely used spatial statistical method for analyzing the spatial distribution of point data. In this study, we employed an extended version of kernel density estimation—weighted kernel density estimation—where we assigned the number of price-negotiated NADs supplied by each drug-providing institution as weights to the spatial points of these hospitals and retail pharmacies. The kernel density value at each point on the map is calculated as:

$$f\left(x\right)=\frac{1}{n}\sum_{i=1}^{n}w_{i}K\left(\frac{\left\|x-x_{i}\right\|}{h}\right)$$

*f(x)*: The kernel density estimate at location *x*, reflecting the density of drug-providing institutions at that location;
*x*: The specific location where the density is estimated, corresponding to the center point of each pixel in the map raster image;
*h*: The radius, representing the coverage of the kernel function;
*x*_*i*_: The drug-providing institution i within a certain radius of point *x*;
*n*: The total number of drug-providing institutions within a certain radius of point *x*;
||*x−x*_*i*_ *|*|: The distance between point *x* and point *x*_*i*_ (Euclidean distance);
*K*: The kernel function, where a quartic kernel function is used in this study;
*w*_*i*_: The weight, which is the number of price-negotiated NADs supplied by the drug-providing institution i.

The World Health Organization (WHO) has recommended a model for assessing health service availability. In this model, the number of healthcare facilities, both public and private, per 10,000 residents is one of the primary indicators. To measure the availability of price-negotiated NADs within a provincial administrative region in China, while considering the drug supplying status of providing institutions, we improved this availability indicator to the weighted supply number of drug-providing institutions (hospitals, retail pharmacies) per 1,000 cancer patients. The formulas are as follows:

$$Weighted\;Supply\;Number\;of\;Drug-Providing\;Hospitals\;{\left(per\;1,000\;patients\right)}_{i}=\frac{1000\times \sum_{j=1}^{n_{Hi}}H_{ij}}{P_{i}}$$

$$Weighted\;Supply\;Number\;of\;Drug-Providing\;Retail\;Pharmacies\;{\left(per\;1,000\;patients\right)}_{i}=\frac{1000\times \sum_{k=1}^{n_{Ri}}R_{ik}}{P_{i}}$$

$$Weighted\;Supply\;Number\;of\;Overall\;Drug-Providing\;Institutions\;{\left(per\;1,000\;patients\right)}_{i}=\frac{1000\times \left(\sum_{j=1}^{n_{Hi}}H_{ij}+\sum_{k=1}^{n_{Ri}}R_{ik}\right)}{P_{i}}$$

*H*_*ij*_: The number of price-negotiated NADs provided by hospital j in province i;
*R*_*ik*_: The number of price-negotiated NADs provided by pharmacy k in province i;
*n*_*Hi*_: The total number of hospitals providing price-negotiated NADs in province i;
*n*_*Ri*_: The total number of retail pharmacies providing price-negotiated NADs in province i;
*P*_*i*_: The number of cancer patients in province i. Since the provincial-level cancer incidence rates cannot be obtained, the estimated number of cancer patients in each province is calculated as the population of the province from the 7th National Census multiplied by the national crude incidence rate for all cancer sites.

### 2.4 Statistical analysis

Our study will explore regional differences in availability across China’s eastern, central, and western regions. Based on socio-economic development and geographic location, the Eastern region includes 11 provinces: Beijing (BJ), Tianjin (TJ), Shanghai (SH), Guangdong (GD), Hainan (HI), Fujian (FJ), Zhejiang (ZJ), Jiangsu (JS), Shandong (SD), Liaoning (LN), Hebei (HE); the central region includes 8 provinces: Anhui (AH), Hubei (HB), Henan (HA), Shanxi (SX), Jilin (JL), Heilongjiang (HL), Jiangxi (JX), Hunan (HN); and the Western region includes 12 provinces: Inner Mongolia (NM), Xinjiang (XJ), Shaanxi (SN), Gansu (GS), Chongqing (CQ), Guangxi (GX), Ningxia (NX), Yunnan (YN), Sichuan (SC), Guizhou (GZ), Qinghai (QH), Tibet (XZ).

#### 2.4.1 The Theil index

The Theil index was applied to evaluate the inequity of the availability. The index is always greater than or equal to zero, with higher values indicating a higher level of inequality. The formula is as follows:

$$T=\frac{1}{n}\sum_{i=1}^{n}\left(\frac{a_{i}}{\bar{a}}\ln\left(\frac{a_{i}}{\bar{a}}\right)\right)$$

*T*: The Theil index;

$\bar{a}$ : The national average availability;
*a*_*i*_: The availability of province i;
*n*: The number of provinces. Due to the absence of data from the Macau Special Administrative Region, Hong Kong Special Administrative Region, and Taiwan Province, this study only analyzes the other 31 provinces.

#### 2.4.2 Global Moran’s index

We will use the global Moran’s Index to reveal whether the availability or unavailability of price-negotiated NADs is spatially clustered. This index measures the degree of spatial autocorrelation of a particular indicator, with values ranging from −1 to +1. A value close to +1 indicates positive spatial autocorrelation (i.e., similar availability values are clustered together), a value close to −1 indicates negative spatial autocorrelation (i.e., similar values are dispersed), and a value close to 0 indicates no significant spatial autocorrelation. The formula is as follows:

$$Global\;Moran^{\prime }s\;I=\frac{n\sum_{i=1}^{n}\sum_{j=1}^{n}W_{ij}\left(a_{i}-\bar{a}\right)\left(a_{j}-\bar{a}\right)}{\sum_{i=1}^{n}\sum_{j=1}^{n}W_{ij}\sum_{i=1}^{n}{\left(a_{i}-\bar{a}\right)}^{2}}$$

$\bar{a}$ : The national average availability;
*a*_*i,*_ *a*_*j*_: The availability of province i and province j;
*W*_*ij*_: Spatial weight matrix element, representing the spatial relationship between province i and province j. If the two provinces are adjacent, *W*_*ij*_ = 1; if they are not adjacent, *W*_*ij*_ = 0;
*n*: The number of provinces.

#### 2.4.3 Regression analysis

To explore the impact of regional economic development and healthcare infrastructure on availability (Gao et al., 2024), we performed multiple linear regression and GWR, an extension of ordinary least squares regression (OLS) that adds a level of modeling sophistication by allowing the relationships between independent and dependent variables to vary by locality (Fotheringham et al., 2002). This model is adept at capturing the local variations in the relationships between variables across space (Ge et al., 2024). The independent variables selected include GDP *per capita* as a measure of economic development and the number of Tertiary A-grade hospitals and the number of chain pharmacies as measures of healthcare infrastructure. The multiple linear regression model (OLS) is as follows:

$$y_{i}=\beta_{0}+\sum_{k}\beta_{k}x_{ik}+\epsilon_{i}$$

*y*_*i*_: The dependent variable, the availability of price-negotiated NADs in province i;
*β*_*0*_: The intercept term;
*x*_*ik*_: The value of the independent variable k (economic development level, healthcare development level) for province i;
*β*_*k*_: The regression coefficient, which reflects the impact of *x*_*ik*_ on the dependent variable;
*ϵ*_*i*_: Error term.

The GWR model expression is:

$$y_{i}=\beta_{0}\left(\mu_{i},v_{i}\right)+\sum_{k}\beta_{k}\left(\mu_{i},v_{i}\right)x_{ik}+\epsilon_{i}$$

*y*_*i*_: The dependent variable, the availability of price-negotiated NADs in province i;

$\mu_{i},v_{i}$ : The centroid coordinates of province i on the map;

$\beta_{0}\left(\mu_{i},v_{i}\right)$ : The intercept term dependent on the coordinates;

$\beta_{k}\left(\mu_{i},v_{i}\right)$ : The coefficient dependent on the coordinates;
*x*_*ik*_: The value of the independent variable k for province i;
*ϵ*_*i*_: Error term.

## 3 Results

### 3.1 Basic status and spatial distribution of price-negotiated NADs supply institutions

#### 3.1.1 Basic status of drug-providing hospitals

On 1 January 2024, a total of 7,322 hospitals were providing at least one national price-negotiated novel anticancer drug, including 3,231 in the eastern region, 2,325 in the central region, and 1,766 in the western region. By October, this number had increased to 7,838 hospitals, with 3,492 in the eastern region, 2,464 in the central region, and 1,822 in the western region. Henan Province had the highest number of drug-providing hospitals in both January and October, while the Tibet Autonomous Region had the fewest. In terms of hospital types, most provinces had the highest number of secondary hospitals providing NADs, followed by tertiary hospitals, with primary hospitals being the least represented.

#### 3.1.2 Basic status of drug-providing retail pharmacies

On 1 January 2024, there were 5,419 retail pharmacies providing at least one price-negotiated NAD, including 2,560 in the eastern region, 1,716 in the central region, and 1,143 in the western region. By October, the total number had increased to 6,098, with 2,850 in the eastern region, 1,962 in the central region, and 1,286 in the western region.

#### 3.1.3 Drug coverage

In January, institutions across all provinces provided an average of 57.78 out of the 71 price-negotiated NADs included in this study. All provinces, except Tibet, covered more than 50 NADs. On average, hospitals provided 6.94 NADs each, while retail pharmacies provided 6.20. By October, provinces were able to cover an average of 67.03 NADs, with most provinces achieving near-complete coverage. Hospitals provided an average of 7.72 NADs each, and retail pharmacies provided 7.33. Detailed data on the basic status of drug-providing institutions are presented in Supplementary Table S2.

#### 3.1.4 Spatial distribution

**FIGURE 1:**
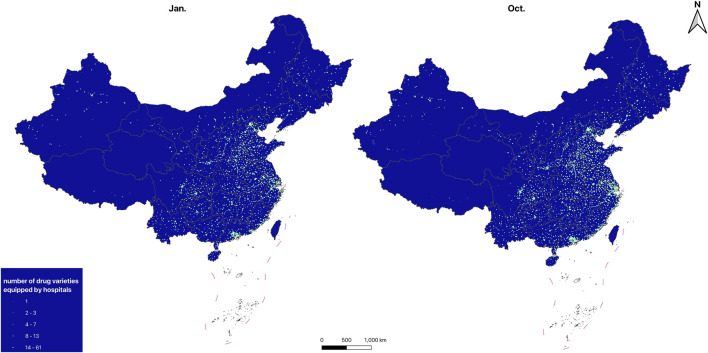
Spatial distribution of drug-providing hospitals.

**FIGURE 2:**
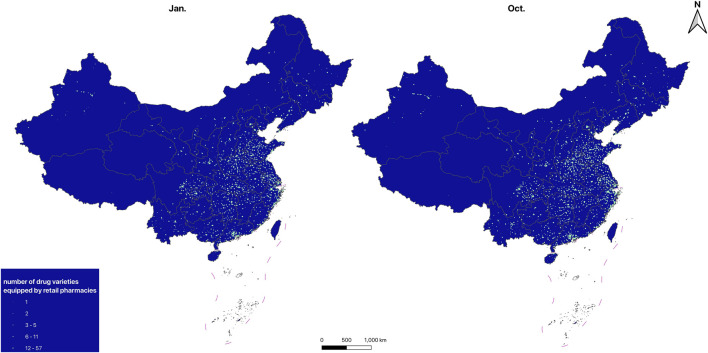
Spatial distribution of drug-providing retail pharmacies.

**FIGURE 3:**
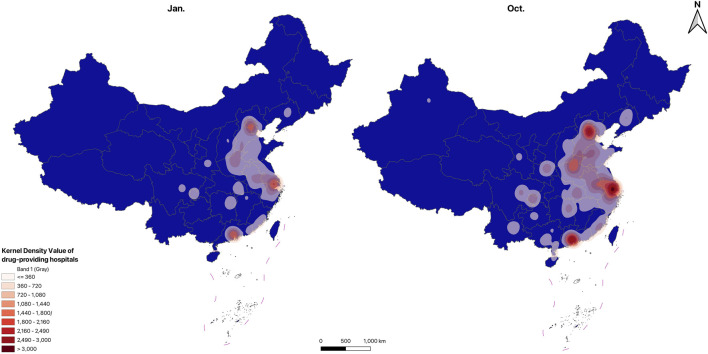
KDE results of drug-providing hospitals.

**FIGURE 4:**
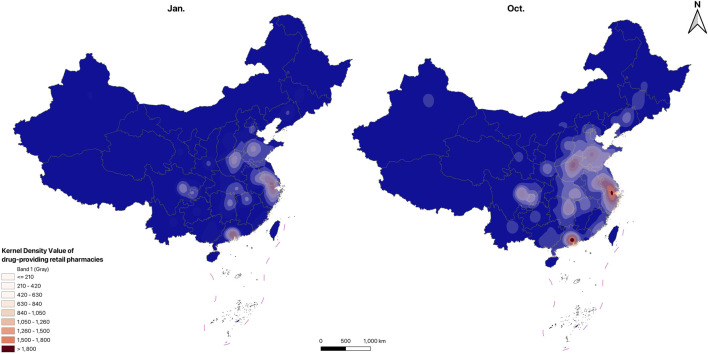
KDE results of drug-providing retail pharmacies.

The distribution maps of drug-providing hospitals and retail pharmacies are shown in Figures 1, 2, the KDE results of drug-providing institutions see in Figures 3, 4. Figures 1, 2 indicate that drug-providing hospitals and retail pharmacies are more concentrated in the Eastern and central regions, with fewer in the Northeast (Liaoning, Jilin, and Heilongjiang) and western regions. The KDE results show a high density of drug-providing hospitals in the Eastern coastal areas, especially around Beijing, the Yangtze River Delta (Jiangsu, Shanghai, and Zhejiang), and the Pearl River Delta (Southern Guangdong), see in Figure 3. While drug-providing pharmacies are concentrated in the Yangtze River Delta and the Pearl River Delta see in Figure 4. From January to October, this distribution pattern became even more pronounced.

### 3.2 Geographical and temporal variations in price-negotiated NADs availability

**FIGURE 5:**
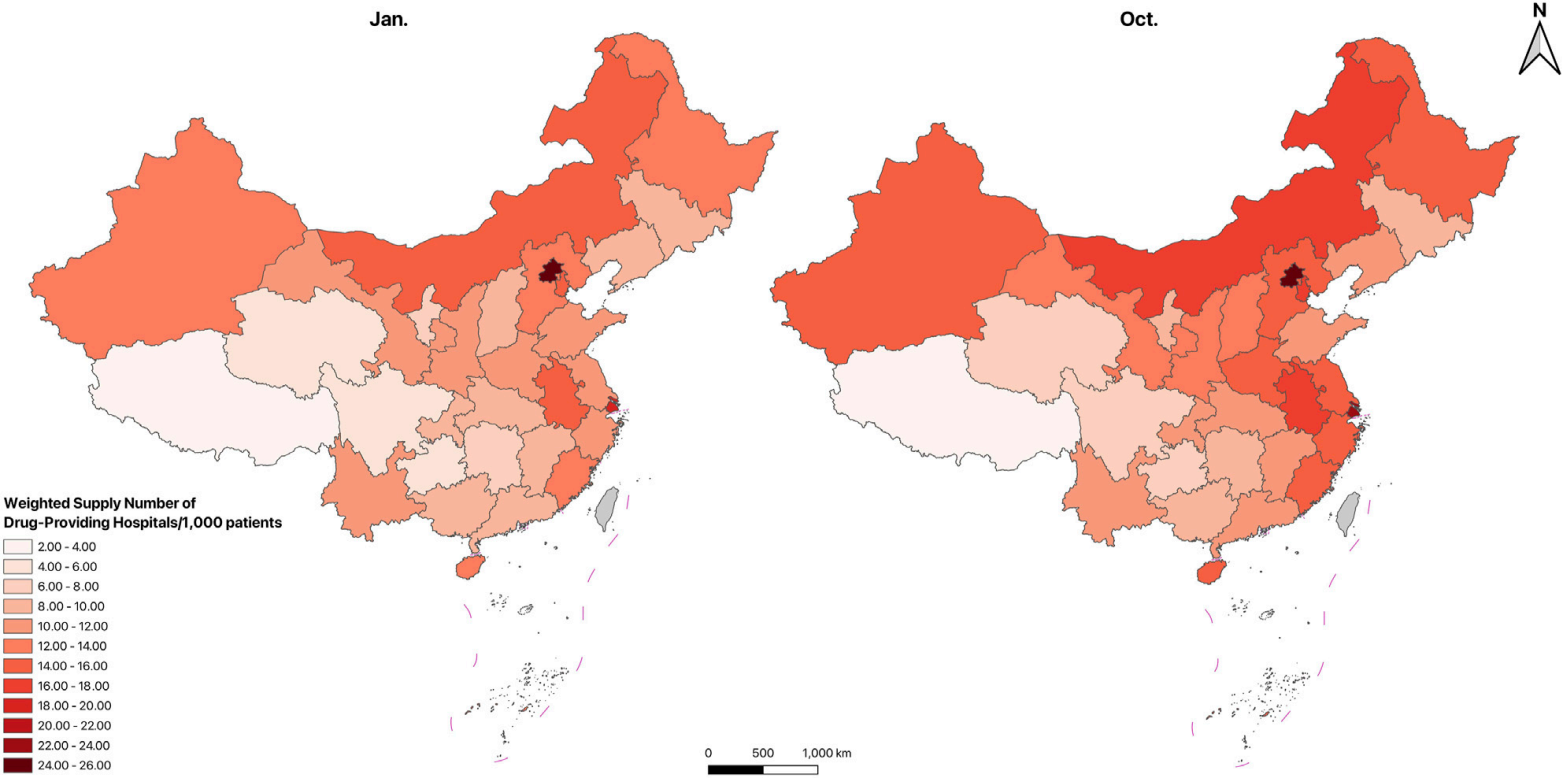
Spatial distribution of price-negotiated NADs availability in hospitals.

**FIGURE 6:**
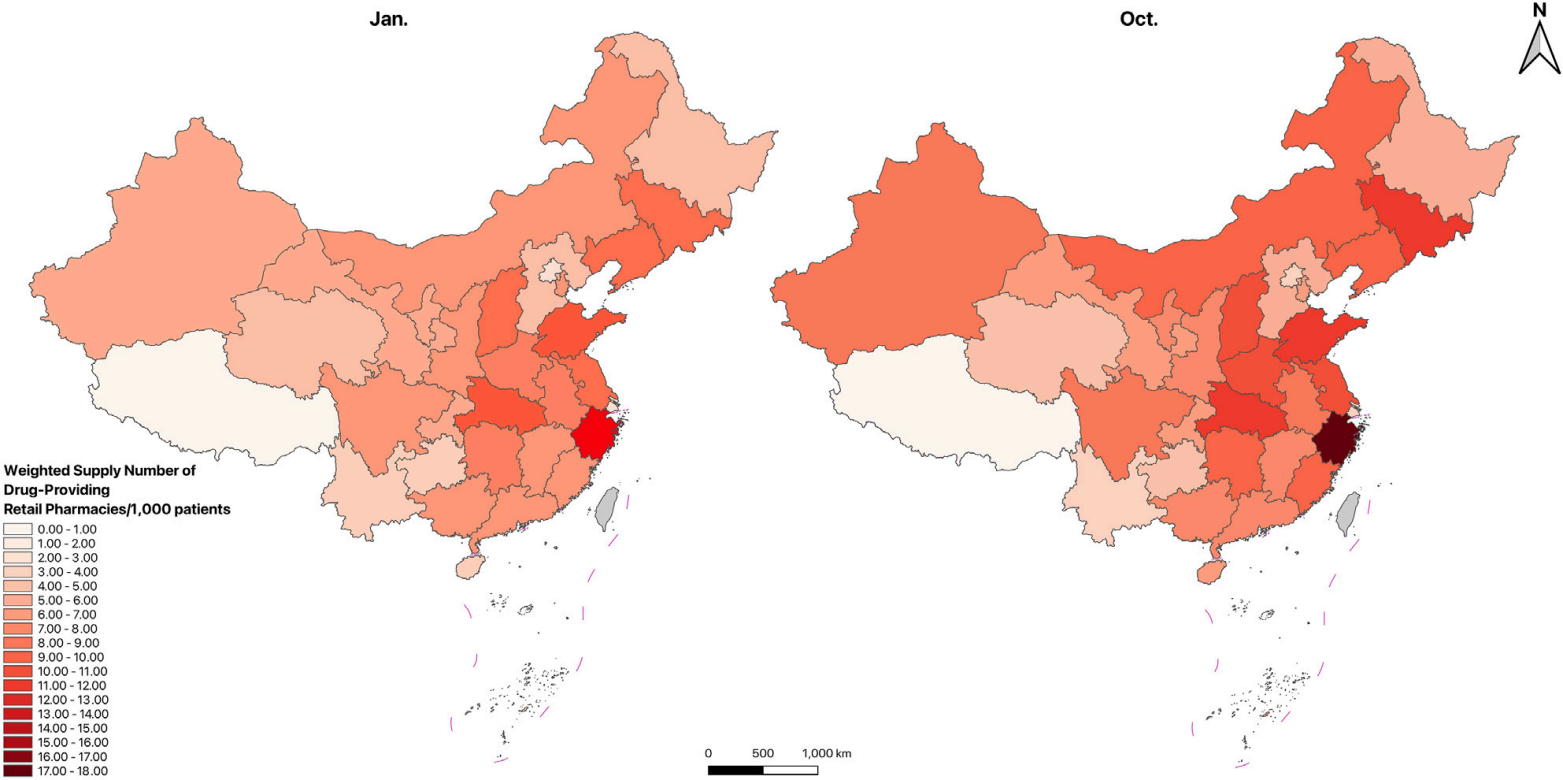
Spatial distribution of price-negotiated NADs availability in retail pharmacies.

**FIGURE 7:**
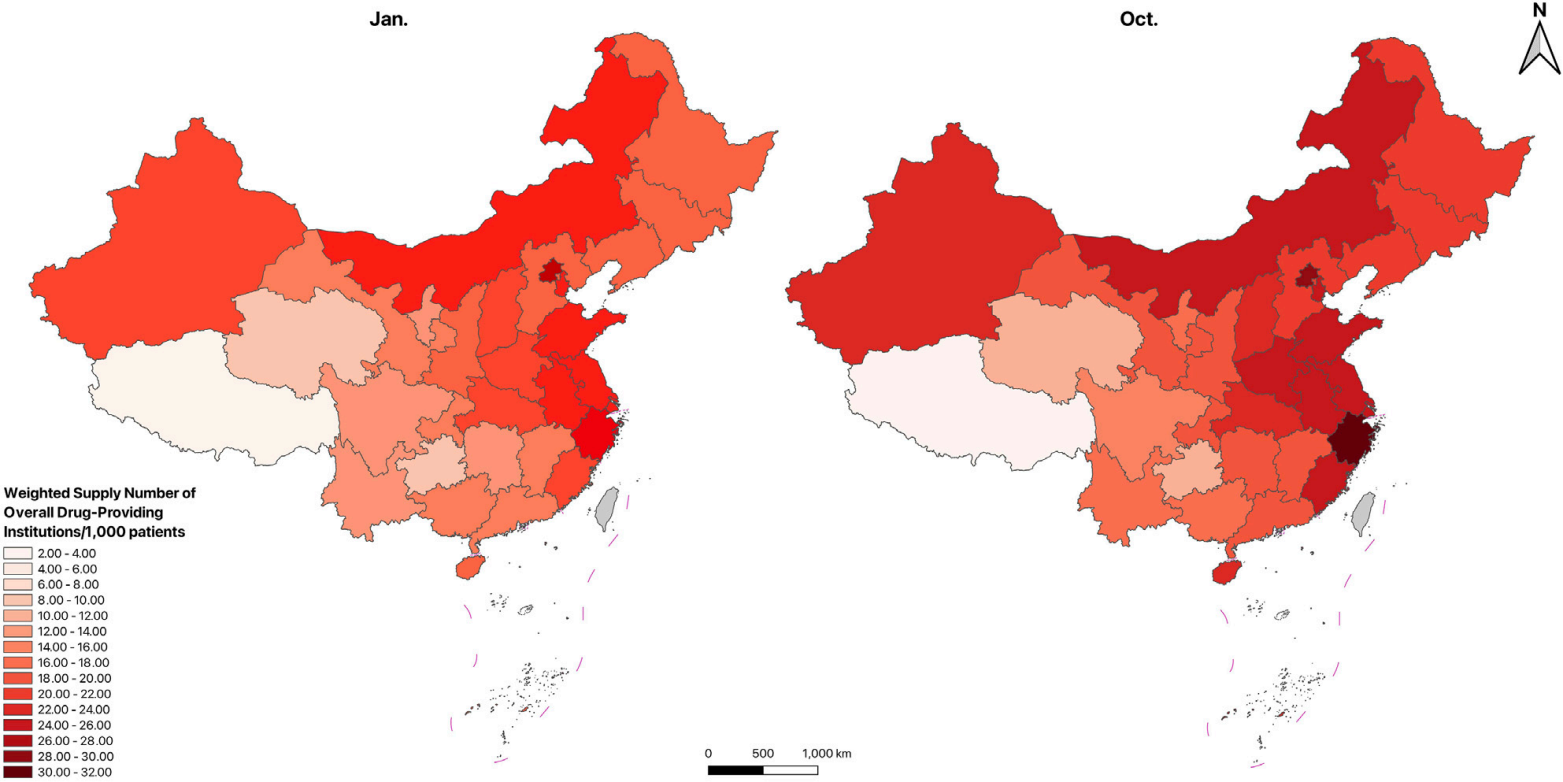
Spatial distribution of price-negotiated NADs availability in overall drug-providing institutions.

Figures 5–7; Supplementary Figures S1–S3 show the spatial distribution and temporal changes of price-negotiated NADs availability in hospitals, retail pharmacies and overall drug-providing institutions across provinces. Hospital-based availability (Figure 5), retail pharmacy-based availability (Figure 6), and overall availability (Figure 7) demonstrate higher levels in the Eastern and central regions, with lower in the Western regions, especially in the Southwest (Sichuan, Guizhou, Yunnan, Qinghai, Tibet). Hospital-based availability was highest in Beijing and Shanghai. While retail pharmacy-based availability was relatively low in Beijing and Shanghai, it peaked in Zhejiang. Overall drug availability was also highest in Beijing. In contrast, hospital-based, retail pharmacy-based and overall availability was the lowest in Tibet. Supplementary Figures S1–S3 indicate that compared to January, the availability significantly improved in October across different institutions and regions. Specific availability results for each province are presented in Table 1.

**TABLE 1:** The availability of price-negotiated NADs.

region	province	weighted supply number of drug-providing hospitals/1000 patients		weighted supply number of retail pharmacies/1000 patients		weighted supply number of overall drug-providing institutions/1000 patients	
		Jan.	Oct.	Jan.	Oct.	Jan.	Oct.
Eastern	Beijing	24.673	25.541	2.286	3.716	26.958	29.257
Eastern	Shanghai	18.636	22.072	2.671	3.541	21.307	25.613
Eastern	Tianjin	14.603	17.157	6.120	7.006	20.723	24.163
Eastern	Hainan	13.381	15.529	3.918	7.140	17.299	22.669
Eastern	Fujian	12.560	14.645	6.783	9.672	19.343	24.316
Eastern	Hebei	12.554	14.746	4.385	5.926	16.939	20.672
Eastern	Jiangsu	11.919	14.557	8.708	11.366	20.627	25.923
Eastern	Zhejiang	11.302	14.266	12.580	17.507	23.883	31.773
Eastern	Shandong	10.171	11.946	9.834	12.485	20.005	24.432
Eastern	Guangdong	9.769	11.699	6.205	7.895	15.974	19.594
Eastern	Liaoning	9.508	11.810	8.066	10.189	17.574	21.998
Central	Anhui	14.226	16.202	7.413	9.427	21.639	25.628
Central	Heilongjiang	12.669	14.552	4.281	5.898	16.950	20.451
Central	Henan	11.573	14.167	7.409	11.388	18.982	25.555
Central	Hubei	9.885	11.390	9.434	12.388	19.319	23.778
Central	Shanxi	9.663	12.110	8.456	10.660	18.119	22.770
Central	Jiangxi	8.865	11.228	6.579	8.269	15.444	19.497
Central	Jilin	8.824	9.943	8.703	12.033	17.527	21.976
Central	Hunan	6.077	8.319	7.570	10.468	13.647	18.787
Western	Inner Mongolia	14.175	16.158	6.716	9.758	20.891	25.916
Western	Xinjiang	12.926	14.793	5.852	9.111	18.778	23.905
Western	Gansu	10.303	12.467	5.544	6.912	15.847	19.379
Western	Shaanxi	10.208	12.155	6.166	7.795	16.374	19.950
Western	Yunnan	10.103	11.820	3.446	4.382	13.549	16.202
Western	Chongqing	9.713	11.310	5.724	6.956	15.437	18.266
Western	Guangxi	8.196	9.912	6.544	8.073	14.740	17.985
Western	Ningxia	7.922	9.425	5.850	7.800	13.772	17.225
Western	Sichuan	5.592	6.585	6.749	8.536	12.341	15.121
Western	Guizhou	5.395	6.640	3.544	4.644	8.939	11.283
Western	Qinghai	4.446	6.076	4.396	5.038	8.842	11.114
Western	Tibet	2.406	2.727	0.080	0.080	2.486	2.807

Table 2 shows the Theil index and Global Moran’s Index. The Theil index of price-negotiated NADs availability in hospitals, pharmacies, and overall drug-providing institutions is relatively low, ranging from 0.044 to 0.093. This indicates that the availability of price-negotiated NADs across provinces is generally balanced, but there are still some differences, retail pharmacy-based availability (0.092, 0.093) is more unequal than hospital-based availability (0.074, 0.062). From January to October, the Theil index for hospital-based availability decreased, suggesting a decline in the inequity of availability between provinces.

**TABLE 2:** The Theil index, and Global Moran’s index of price-negotiated NADs availability.

Availability indicators	The theil index	Moran’s I (*p* value)
Weighted Supply Number of Drug-Providing Hospitals/1,000 patients		
Jan.	0.074	0.315 (*p*<0.01)
Oct.	0.062	0.362 (*p*<0.001)
Weighted Supply Number of Drug-Providing Retail Pharmacies/1,000 patients		
Jan.	0.092	0.104 (*p =* 0.241)
Oct.	0.093	0.103 (*p =* 0.243)
Weighted Supply Number of Overall Drug-Providing Institutions/1,000 patients		
Jan.	0.045	0.452 (*p<*0.0001)
Oct.	0.044	0.453 (*p<*0.0001)

Global Moran’s Index of price-negotiated NADs availability in hospitals and overall drug-providing institutions was greater than 0, with a *p-*value lower than 0.01, indicating a spatial positive autocorrelation or clustering, From January to October, this spatial autocorrelation strengthened, particularly in hospitals (Moran’s I of hospital-based availability 0.315–0.362, overall availability 0.452–0.453). However, this spatial autocorrelation was not pronounced in retail pharmacy-based availability (*p >* 0.05).

### 3.3 The impact of reginal economic development/healthcare infrastructure on price-negotiated NADs availability

#### 3.3.1 Independent variables

We constructed a total of 12 models, including both OLS and GWR models, for price-negotiated NADs availability in hospitals, retail pharmacies, and overall drug-providing institutions (in January and October). In these models, we examined the relationship between the availability and regional economic development (measured by GDP *per capita* 2023, unit: ten thousand yuan) as well as regional healthcare infrastructure (measured by the number of tertiary A-grade hospitals, unit: hospitals; and number of chain pharmacies, unit: thousand pharmacies). Due to collinearity between the two independent variables representing healthcare infrastructure (variance inflation factor >0.5), we determined the most appropriate variable for each model based on the type of institution and model fit (R^2^). Specifically, we used the number of tertiary A-grade hospitals to assess its impact on hospital-based availability and overall availability, while the number of chain pharmacies was used to assess its impact on retail pharmacy-based availability, process of independent variable selection is presented in Supplementary Material.

#### 3.3.2 Regression analysis results

The GWR was better at explaining the association between independent variables and price-negotiated NADs availability than the OLS model. For example, the OLS model for hospital-based availability (Jan.) yielded a lower adjusted R^2^ = 0.462 compared with an adjusted R^2^ = 0.580 for the GWR model, The same pattern was observed in the other models (see Supplementary Table S3).

The results of the OLS and GWR models are shown in Tables 3, 4. A positive, statistically significant and strengthened association between hospital-based availability and GDP *per capita* was observed in both the OLS (Jan., coef = 0.787; 95%CI = 0.481 to 1.093; *p* < 0.0001; Oct., coef = 0.833; 95%CI = 0.508 to 1.159; *p* < 0.0001) and the GWR model [Jan., Mean coef (SD) = 0.795 (0.047); Range = 0.725 to 0.904; Oct., Mean coef (SD) = 0.834 (0.044); Range = 0.752–0.927]. As shown in Supplementary Figure S4 the GWR model indicates that this association exhibits a higher correlation in the West and a lower correlation in the East. Moreover, this association becomes stronger over time across all regions, see in Supplementary Figure S5.

**TABLE 3:** OLS results.

Dependent Variable	Independent Variable	coef (95%CI)	SE	*p* value
Weighted Supply Number of Drug-Providing Hospitals/1,000 patients	GDP per capita 2023			
	Jan.	0.787 (0.481, 1.093)	0.150	<0.0001
	Oct.	0.833 (0.508, 1.159)	0.159	<0.0001
	Number of Tertiary A-Grade Hospitals			
	Jan.	−0.007 (-0.045, 0.032)	0.019	0.724
	Oct.	−0.006 (-0.047, 0.035)	0.020	0.775
Weighted Supply Number of Drug-Providing Retail Pharmacies/1,000 patients	GDP per capita 2023			
	Jan.	0.020 (-0.214 0.255)	0.114	0.861
	Oct.	0.038 (−0.282, 0.359)	0.157	0.808
	Number of Chain Pharmacies			
	Jan.	0.098 (0.012, 0.184)	0.042	<0.05
	Oct.	0.122 (0.005, 0.240)	0.057	<0.05
Weighted Supply Number of Overall Drug- Providing Institutions/1,000 patients	GDP per capita 2023			
	Jan.	0.744 (0.378, 1.111)	0.179	<0.001
	Oct.	0.794 (0.323, 1.26)	0.230	<0.01
	Number of Tertiary A-grade hospitals			
	Jan.	0.031 (-0.015, 0.077)	0.022	0.174
	Oct.	0.042 (-0.017, 0.101)	0.029	0.160

**TABLE 4:** GWR results.

Dependent variable	Independent variable	Mean coef	Range	SD
Weighted Supply Number of Drug-Providing Hospitals/1,000 patients	GDP per capita 2023			
	Jan.	0.795	0.725 to 0.904	0.047
	Oct.	0.834	0.752 to 0.927	0.044
	Number of Tertiary A-Grade Hospitals			
	Jan.	−0.014	−0.023 to 0.023	0.010
	Oct.	−0.014	−0.024 to 0.024	0.011
Weighted Supply Number of Drug-Providing Retail Pharmacies/1,000 patients	GDP per capita 2023			
	Jan.	−0.002	−0.107 to 0.198	0.080
	Oct.	0.014	−0.138 to 0.293	0.110
	Number of Chain retail pharmacies			
	Jan.	0.084	0.078 to 0.108	0.006
	Oct.	0.107	0.087 to 0.145	0.010
Weighted Supply Number of Overall Drug- Providing Institutions/1,000 patients	GDP per capita 2023			
	Jan.	0.726	0.554 to 0.973	0.119
	Oct.	0.763	0.531 to 1.107	0.161
	Number of Tertiary A-Grade Hospitals			
	Jan.	0.020	0.000 to 0.096	0.029
	Oct.	0.018	−0.002 to 0.122	0.022

Such a pattern of correlation (positive, significant, GWR coef higher in the West) was also observed between retail pharmacy-based availability and the number of chain pharmacies [OLS: Jan., coef = 0.098; 95%CI = 0.012 to 0.184; *p* < 0.05; Oct., coef = 0.122; 95%CI = 0.005 to 0.240; *p* < 0.05; GWR: Jan., Mean coef (SD) = 0.084 (0.006); Range = 0.078 to 0.108; Oct., Mean coef (SD) = 0.107 (0.010); Range = 0.087–0.145], see in Supplementary Figure S10.

Likewise, a similar positive, statistically significant relationship between overall availability and GDP *per capita* was found in both the OLS (Jan., coef = 0.744; 95%CI = 0.378 to 1.111; *p* < 0.001; Oct., coef = 0.794; 95%CI = 0.323 to 1.26; *p* < 0.01) and GWR model [Jan., Mean coef (SD) = 0.726 (0.119); Range = 0.554 to 0.973; Oct., Mean coef (SD) = 0.763 (0.161); Range = 0.531–1.107]. As shown in Supplementary Figure S12, the GWR model indicates that this association also exhibits a higher and strengthened correlation in the West.

Where the OLS model estimated that the association between overall availability and number of tertiary A-grade hospitals was not significant (*p* > 0.05), the GWR model observed a positive relationship in some western provinces (coef>0), see in Supplementary Figure S14.

Finally, between hospital-based availability and the number of tertiary-A grade hospitals, as well as between retail pharmacy-based availability and GDP *per capita*, not only did the OLS model fail to observe a significant correlation *(p* > 0.05), but the regression coefficients of the GWR models across provinces were also within a range close to 0. This indicates that the number of tertiary A-grade hospitals has little impact on hospital-based availability, and GDP *per capita* has little impact on retail pharmacy-based availability, with spatial stability. For details, see Tables 3, 4, and other figures in Supplementary Material.

## 4 Discussion

This study analyzes the geographical variations of the availability of price-negotiated NADs nationwide using GIS and spatial statistical methods, as well as the changes in these variations over time and their determining factors. To our knowledge, this is the first study to assess drug availability across China using spatial methods. The results show that the availability of price-negotiated NADs in hospitals and overall drug-providing institutions exhibit a higher level in the Eastern region and a lower level in the Western region, supporting the findings of similar studies (Guan et al., 2018; Gao et al., 2024). Additionally, our study found spatial autocorrelation for both hospital-based availability and overall availability, with higher availability in Beijing, Shanghai, and surrounding provinces, and lower availability in southwest provinces such as Guizhou and Tibet, reflecting a clear regional imbalance. Through regression analysis, we confirmed previous studies’ speculations that inequality in access to anticancer drugs may be related to socio-economic factors and regional healthcare infrastructure differences (Gao et al., 2024). Moreover, we found that this correlation is spatially unstable: in the Western regions, economic development and healthcare infrastructure have a greater impact on the availability of price-negotiated NADs.

### 4.1 Geographic disparities: regional imbalances in the triple chain of infrastructure, economic development, and policy coordination capacity

National price-negotiated NADs, characterized by stringent clinical usage criteria, high costs, and supply chains closely tied to the healthcare security administration system, inherently possess a tripartite nature—medical, commercial, and policy-driven. This unique combination intrinsically links their regional availability to local healthcare infrastructure, levels of economic development, and the capacity for policy coordination.

#### 4.1.1 Infrastructure

First, many NADs rely on cold-chain logistics. Eastern coastal regions benefit from well-developed ports, airports, and pharmaceutical cold-chain infrastructure, whereas the Southwest is characterized by complex terrain and poor transportation networks. Second, NADs are subject to highly stringent diagnostic and usage protocols, their availability heavily depends on top-tier tertiary hospitals and specialized oncology institutions which are densely located in eastern China. Lastly, the eastern region enjoys more advanced digital infrastructure, such as electronic prescription circulation systems and integrated medical insurance settlement platforms, which can enhance the responsiveness of supply chains and eliminate reimbursement barriers (Feng et al., 2025).

#### 4.1.2 Economic development

The pharmaceutical industry is highly concentrated in the eastern coastal regions. These enterprises are more likely to prioritize their home regions for market expansion. Additionally, the high prices of NAD place financial pressure not only on patients but also on local healthcare security funds., Underdeveloped areas may struggle more with ensuring the supply of these high-cost drugs.

#### 4.1.3 Policy coordination

Regional authorities need to balance cost-containment efforts under DRG/DIP models with the policy goal of ensuring access to innovative drugs (Feng et al., 2024). In this regard, eastern coastal provinces generally demonstrate stronger implementation capacity compared to western regions (Healthcare Security Administration of Zhejiang province and Health Commission of Zhejiang province, 2021; Healthcare Security Administration of Guangdong Province, 2024; Healthcare Security Administration of Shanghai, 2024; National healthcare Security Administration, 2024b).”

### 4.2 Retail pharmacy-based availability: the “dual-channel” policy and low provision in Beijing and Shanghai

This study also addresses the issue of the lack of retail pharmacy data in existing studies on the availability of price-negotiated drugs (Zhu et al., 2022; Gao et al., 2024; Zhang et al., 2024). In 2021, the government implemented a “dual-channel” management policy to improve the availability of price-negotiated drugs, allowing patients to obtain the drugs from retail pharmacies in addition to hospitals (Gao et al., 2024). We found that the availability of price-negotiated NADs in retail pharmacies is also higher in the Eastern and central regions, while the Southwest region remains low. Although retail pharmacies are an important supplementary channel for the supply of price-negotiated drugs (Yang et al., 2023), drug-providing retail pharmacies may face issues such as inadequate cold chain distribution and inconsistent patient medication guidance. Additionally, high-priced drugs like NADs are facing increasingly stringent external prescription management, so hospitals remain the main supplier of price-negotiated NADs. We found that in regions where the retail pharmacy-based availability is relatively low, such as Beijing and Shanghai, hospital-based availability and overall availability are very high, reflecting these economically developed provinces’ strong focus on ensuring the availability of price-negotiated NADs in hospitals. Furthermore, retail pharmacy-based availability shows no spatial clustering or correlation with regional economic development, only with the number of chain pharmacies. This is likely because supplying these drugs requires applying to the government for sales and medical insurance reimbursement qualifications, and provinces with more chain pharmacies tend to have more retail pharmacies that meet the qualifications.

### 4.3 Temporal variations: inequality remains unresolved

Our study found that, 9 months after the 2023 price negotiation, the availability of NADs significantly improved nationwide. However, we also observed that the imbalance and spatial autocorrelation of availability between provinces still exists, especially for hospital-based availability. For example, the availability in the adjacent eastern coastal provinces such as Shanghai, Jiangsu, Zhejiang, and Fujian remain high, while provinces in the Southwest such as Tibet, Sichuan, Guizhou, Yunnan, and Qinghai similarly show lower availability, which still needs attention.

### 4.4 Policy recommendations

Efforts should focus on addressing the variations in the availability of anticancer drug treatment across different regions, ensuring that patients nationwide have equitable access to innovative drugs. First, the primary responsibility of hospitals for supplying negotiated drugs should be firmly enforced. Provincial-level authorities should continuously improve performance evaluation mechanisms, implement refined and separate management and reimbursement policies for price-negotiated drugs, and exclude them from assessment indicators such as drug proportion and average cost per visit. Second, the “dual-channel” management mechanism should be further enhanced by establishing clearer criteria for selecting designated retail pharmacies—considering their geographic proximity to hospitals. Developing a unified online prescription transfer platform. would also improve the efficiency, transparency, and safety of prescription circulation. Additionally, incentive mechanisms should be introduced to encourage retail pharmacies to handle external prescriptions and supply innovative drugs.

The country needs to optimize the allocation of high-quality medical resources while prioritizing economically underdeveloped areas to guarantee fair access to high-quality and affordable NADs for all cancer patients, regardless of their location. In the short term, the government should encourage developed cities in the Southwest or advanced medical institutions in the Southeast coastal regions to support areas such as Tibet and Qinghai, helping to establish regional medical centers capable of bearing the responsibility for local cancer diagnosis and treatment. At the same time, pharmaceutical manufacturers and distribution companies should be incentivized to allocate more resources toward less developed southwestern regions and to set up regional drug distribution hubs. In the long term, efforts may include expanding public transportation networks to reduce the time cost for patients in remote areas to access medication, and enhancing public education to improve cancer screening coverage, thereby reducing the future burden of anticancer drug demand. These actions, along with continued support for pharmaceutical innovation, should remain a central concern for health policymakers.

## 5 Limitations

This study also has some limitations. First, our assessment of availability only considered the number of drug-providing institutions and their supply capacity, neglecting barriers to patients’ actual access to drugs, such as transportation and time constraints, which may have led to an overestimation of drug availability in sparsely populated provinces. Second, our study was conducted at the provincial administrative level, which may overlook internal differences within provinces. Lastly, we only explored the correlation between economic development levels, healthcare infrastructure, and the availability of price-negotiated NADs. Future research should further explore other factors contributing to this geographical variation, such as policies and disease incidence.

## 6 Conclusion

The availability of price-negotiated NADs is spatially clustered in the Eastern provinces, while it is lower in the Southwest, particularly in hospitals. This distribution pattern is related to local economic development and healthcare infrastructure, and this clustering and correlation have strengthened over time after price negotiation.

## Data Availability

Publicly available datasets were analyzed in this study. This data can be found here: https://wx.nhsa.gov.cn/#/pages/negotiatingDrugs/negotiatingDrugs.
