# Supplementary material for: Geographical and temporal variations in availability of national price negotiated novel anticancer drugs: a spatial statistical study based on two cross-sectional datasets in China
Source: Front Pharmacol. 2025 Jul 25;16:1604008. doi: 10.3389/fphar.2025.1604008 (PMC12331625; doi:10.3389/fphar.2025.1604008)
Supplement: Supplementary file 1 [file Supplementaryfile1.docx]

Supplementary Material

# Drugs under evaluation

All 71 NADs that underwent the 2023 National Drug Price Negotiation were included in the study, including 49 small molecule inhibitors, 15 antibodies, and 7 hormones, as detailed in Supplementary Table 1. Process of independent variable selection

Supplementary Table 1, 71 NADs included in National Reimbursement Drug List (2024.01)

| Novel anticancer drugs | Mechanism of Action | Indication (Cancer Site) |
| --- | --- | --- |
| **Small molecule inhibitors** |  |  |
| Abemaciclib | CDK4/6 Inhibitor | Female breast |
| Acalabrutinib | BTK Inhibitor | Lymphoma |
| Alectinib | ALK Inhibitor | Lymphoma |
| Almonertinib Mesilate | EGFR Inhibitor | Lung |
| Anlotinib | VEGFR Inhibitor | Lung |
| Apatinib Mesylate | VEGFR2 Inhibitor | Pancreas |
| Avapritinib | KIT/PDGFRA Inhibitor | Stomach |
| Befotertinib Mesylate | EGFR-TKI Inhibitor | Lung |
| Brigatinib | ALK/EGFR Inhibitor | Lung |
| Carfilzomib | Proteasome Inhibitor | Multiple myeloma |
| Chidamide | HDAC Inhibitor | Lymphoma |
| Crizotinib | ALK Inhibitor | Lymphoma |
| Dabrafenib mesylate | B/CRAF Inhibitor | Melanoma of skin |
| Dacomitinib | EGFR/HER2/HER4 Inhibitor | Lung |
| Dalpiciclib isethionate | CDK Inhibitor | Female breast |
| Donafenib Tosilate | Raf/PDGFR/VEGFR Inhibitor | Liver |
| Duvelisib | PI3γ/δ Inhibitor | Lymphoma |
| Ensartinib | ALK Inhibitor | Lymphoma |
| Entrectinib | ALK/ROS1/TrkA Inhibitor | Lung |
| Everolimus | mTOR Inhibitor | Kidney |
| Flumatinib Mesylate | Bcr-Abl Inhibitor | Leukemia |
| Fluzoparib | PARP Inhibitor | Ovary |
| Fruquintinib | VEGFR1/2/3 Inhibitor | Colon-rectum |
| Furmonertinib Mesilate | EGFR Inhibitor | Lung |
| Glumetinib | c-Met Inhibitor | Lung |
| Icotinib Hydrochloride | EGFR Inhibitor | Lung |
| Iruplinalib | ALK Inhibitor | Lymphoma |
| Linperlisib | PI3Kδ Inhibitor | Lymphoma |
| Lorlatinib | ALK Inhibitor | Lung |
| Neratinib Maleate | HER2 Inhibitor | Female breast |
| Olverembatinib | Bcr-Abl Inhibitor | Leukemia |
| Orelabrutinib | BTK Inhibitor | Lymphoma |
| Osimertinib Mesylate | EGFR Inhibitor | Lung |
| Pamiparib | PARP Inhibitor | Ovary |
| Pyrotinib Maleate | HER2 Inhibitor | Female breast |
| Ribociclib Succinate | CDK4/6 Inhibitor | Female breast |
| Ripretinib | PDGFR/c-Kit Inhibitor | Stomach |
| Ruxolitinib Phosphate | JAK Inhibitor | Multiple myeloma |
| Savolitinib | c-Met Inhibitor | Lung |
| Selinexor | XPO1 Inhibitor | Multiple myeloma |
| Selumetinib Hydrogen Sulfate | MEK1/2 Inhibitor | Brain, CNS |
| Sonidegib Phosphate | SMO Inhibitor | Basal cell carcinoma |
| Surufatinib | VEGFR1/2/3 Inhibitor | Pancreas |
| Toluenesulfonate Nilaparil | RARP1/2 Inhibitor | Ovary |
| Trametinib | MEK Inhibitor | Melanoma of skin |
| Vemurafenib | BRAF Inhibitor | Melanoma of skin |
| Venetoclax | BCL-2 Inhibitor | Leukemia |
| Vorolanib | VEGFR Inhibitor | Kidney |
| Zanubrutinib | BTK Inhibitor | Lymphoma |
|  |  |  |
| **Antibodies** |  |  |
| Camrelizumab | anti-PD-1 mAb | Lymphoma |
| Cetuximab | anti- IgG1 mAb | Colon-rectum |
| Daratumumab | anti-CD38 mAb | Multiple myeloma |
| Disitamab vedotin | anti-HER2 ADC | Stomach |
| Inetetamab | anti-HER2 mAb | Female breast |
| Nimotuzumab | anti-EGFR mAb | Nasopharynx |
| Obinutuzumab | anti-CD20 mAb | Lymphoma |
| Polatuzumab Vedotin | anti-CD79B ADC | Lymphoma |
| Ripertamab | anti-CD20 mAb | Lymphoma |
| Sintilimab | anti-IgG4 mAb | Lymphoma |
| Tislelizumab | anti-IgG4 mAb | Lymphoma |
| Toripalimab | anti-IgG4 mAb | Melanoma of skin |
| Trastuzumab | anti-HER2 ADC | Female breast |
| Trastuzumab Emtansine | anti-HER2 ADC | Female breast |
| Zuberitamab | anti-CD20 mAb | Lymphoma |
|  |  |  |
| **Hormones** |  |  |
| Apalutamide | hormone therapy | Prostate |
| Darolutamide | hormone therapy | Prostate |
| Degarelix Acetate | hormone therapy | Prostate |
| Goserelin | hormone therapy | Prostate |
| Goserelin Acetate | hormone therapy | Prostate |
| Rezvilutamide | hormone therapy | Prostate |
| Triporelin Acetate | hormone therapy | Prostate |

# Process of independent variable selection: collinearity and model fit test

In the process of constructing the OLS models, we performed independent variable selection based on the real-world meaning of the independent variables, the results of the collinearity test, and the goodness-of-fit calculation, as detailed in Supplementary Table 2 (Excel). Since both the number of tertiary A-grade hospitals and the number of chain pharmacies reflect the level of regional healthcare infrastructure, we constructed models A to D based on the type of institutions in the dependent variable, and these models passed the collinearity test. However, when we attempted to include both the number of tertiary A-grade hospitals and the number of chain pharmacies in the same model to assess their combined impact on the overall availability dependent variable, as shown in models E and H, we identified collinearity between them. Therefore, we eliminated one of them step by step and constructed models F, G, and I, J. By comparing the Adjusted R², we retained models G and I, which had better goodness-of-fit, as indicated by the cells filled in green. Finally, based on the results of the OLS models, we constructed the corresponding GWR models.

# Temporal changes of price-negotiated NADs availability

#
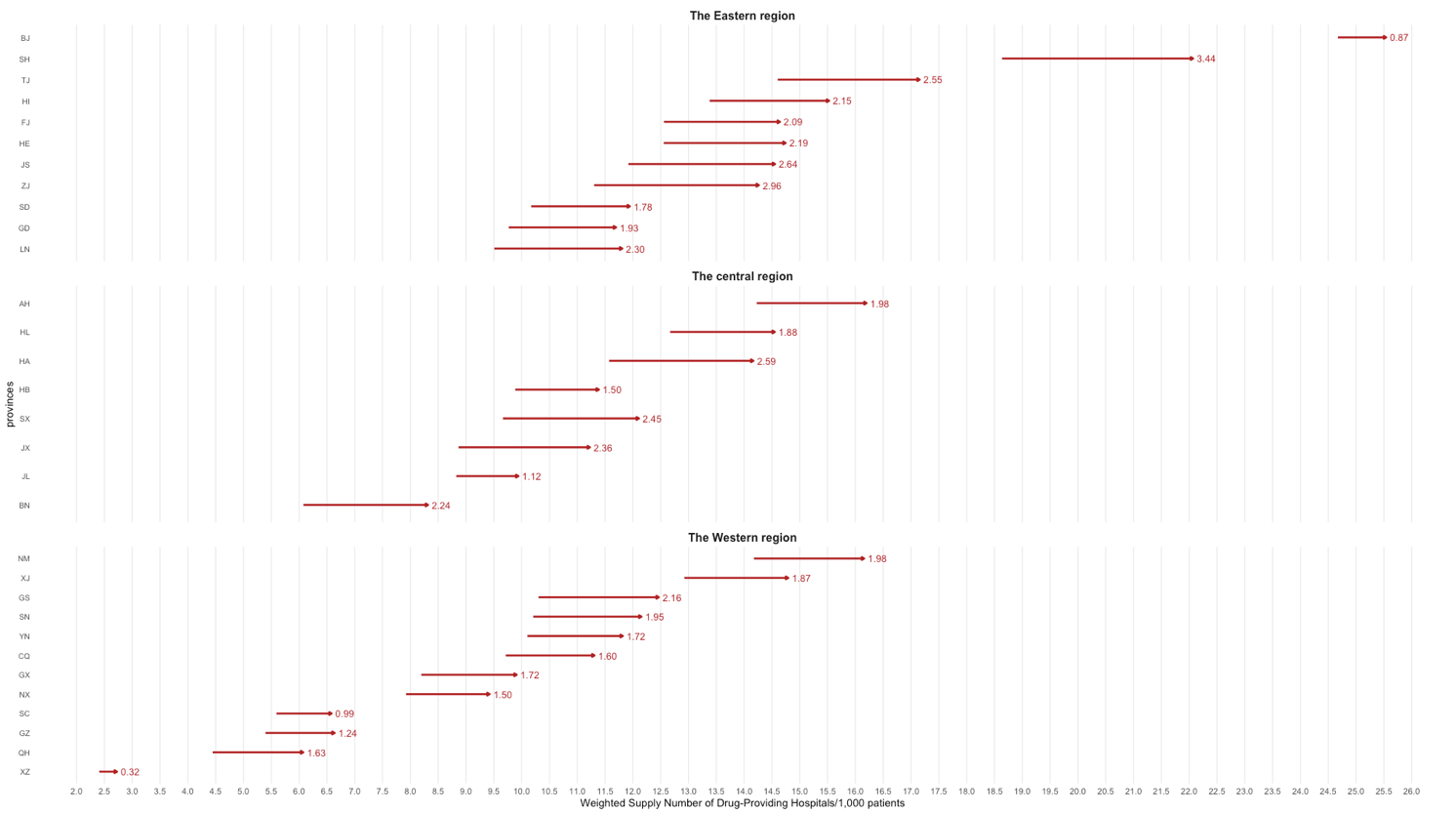


Supplementary Figure 1, Temporal changes of price-negotiated NADs availability in hospitals


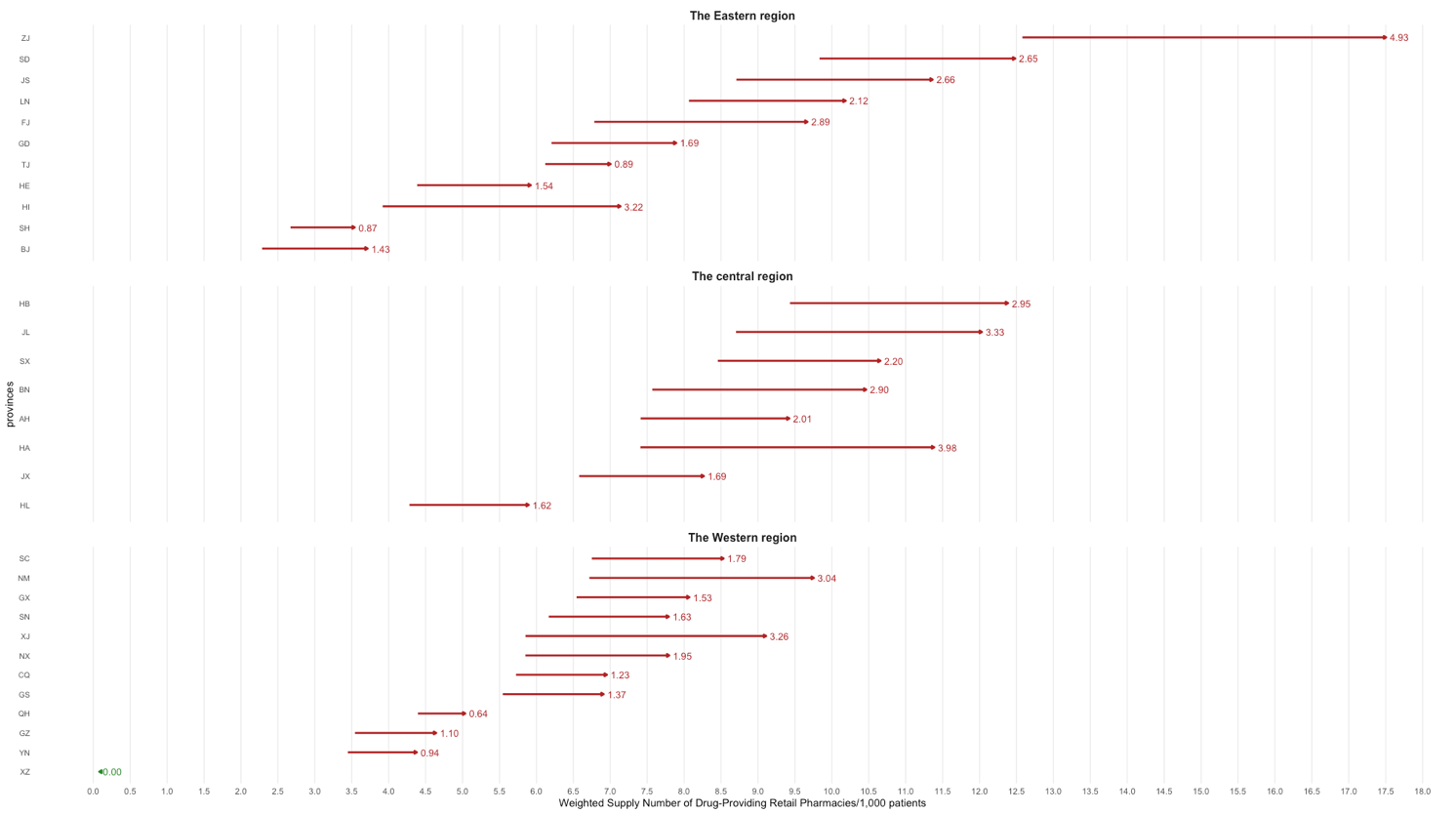


Supplementary Figure 2, Temporal changes of price-negotiated NADs availability in retail pharmacies


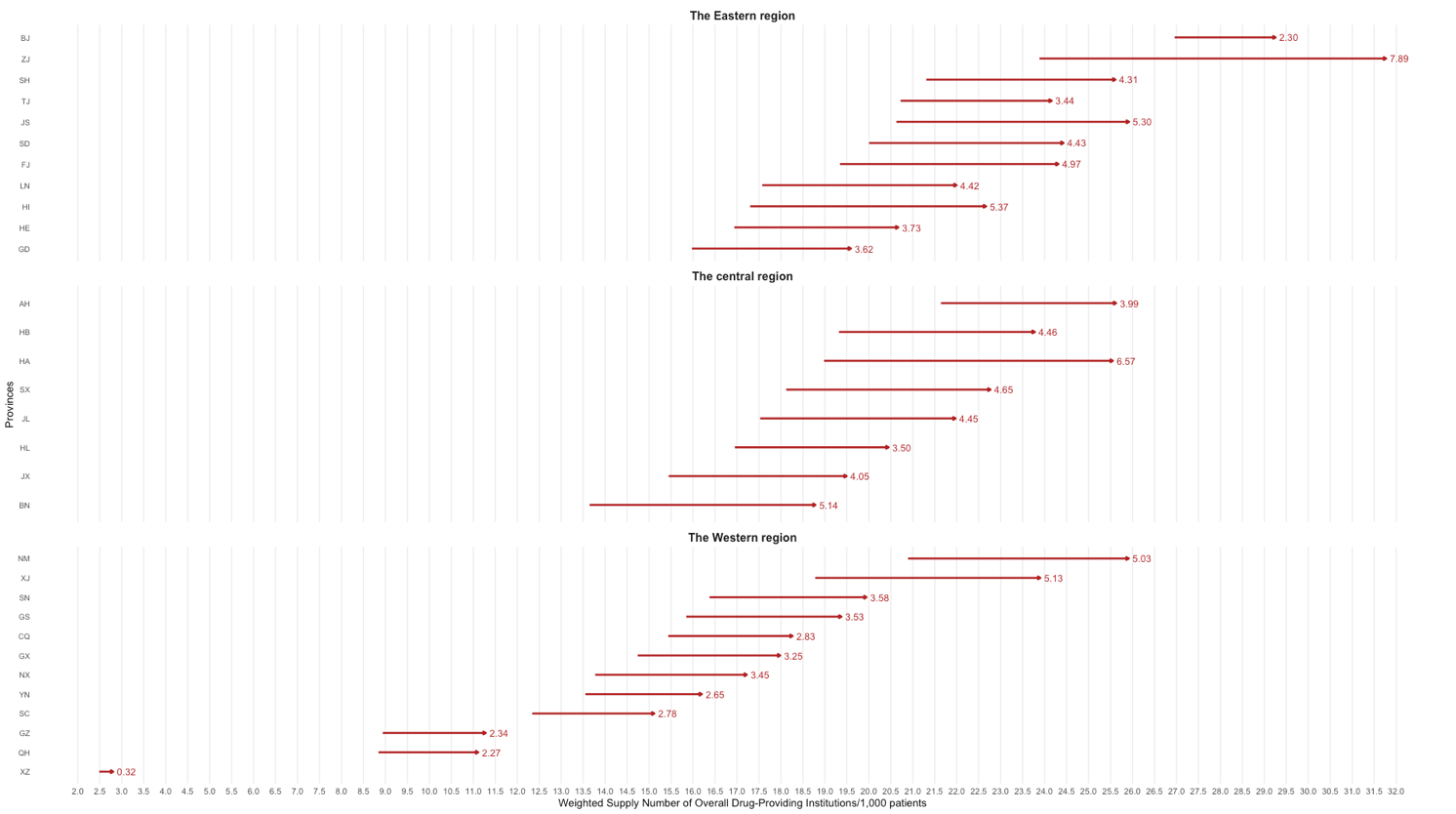


Supplementary Figure 3, Temporal changes of price-negotiated NADs availability in overall drug-providing institutions

# GWR results and their temporal changes


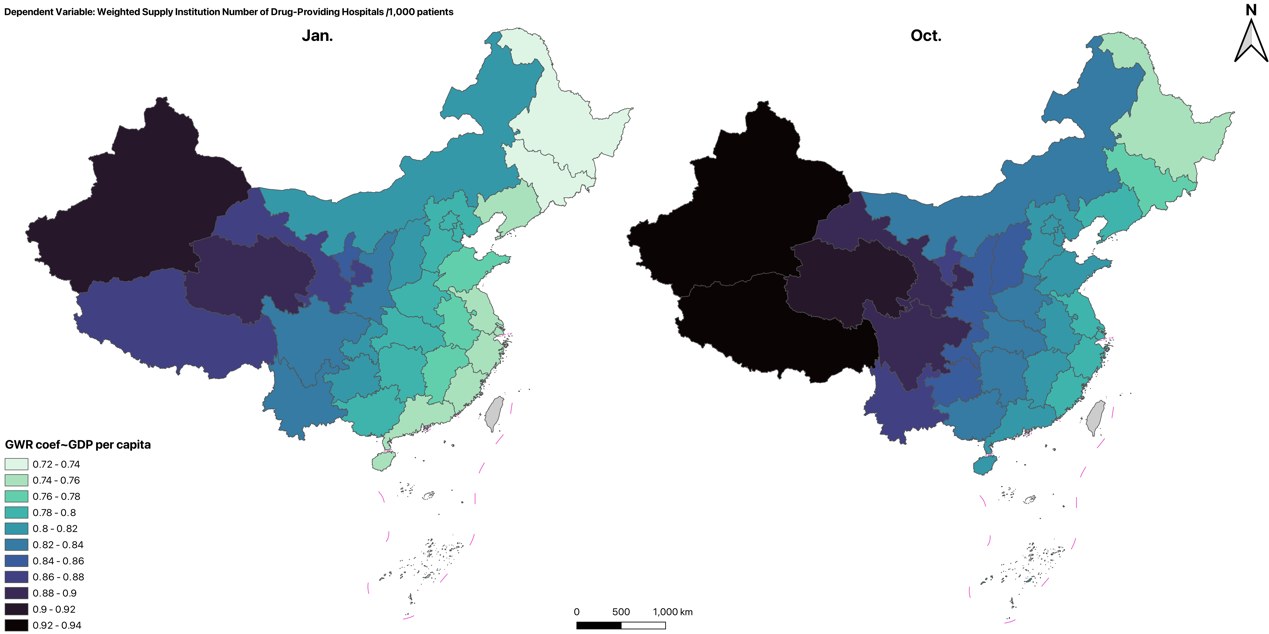


Supplementary Figure 4, GWR result (Dependent variable, hospital-based availability; Independent variable, GDP per capita)


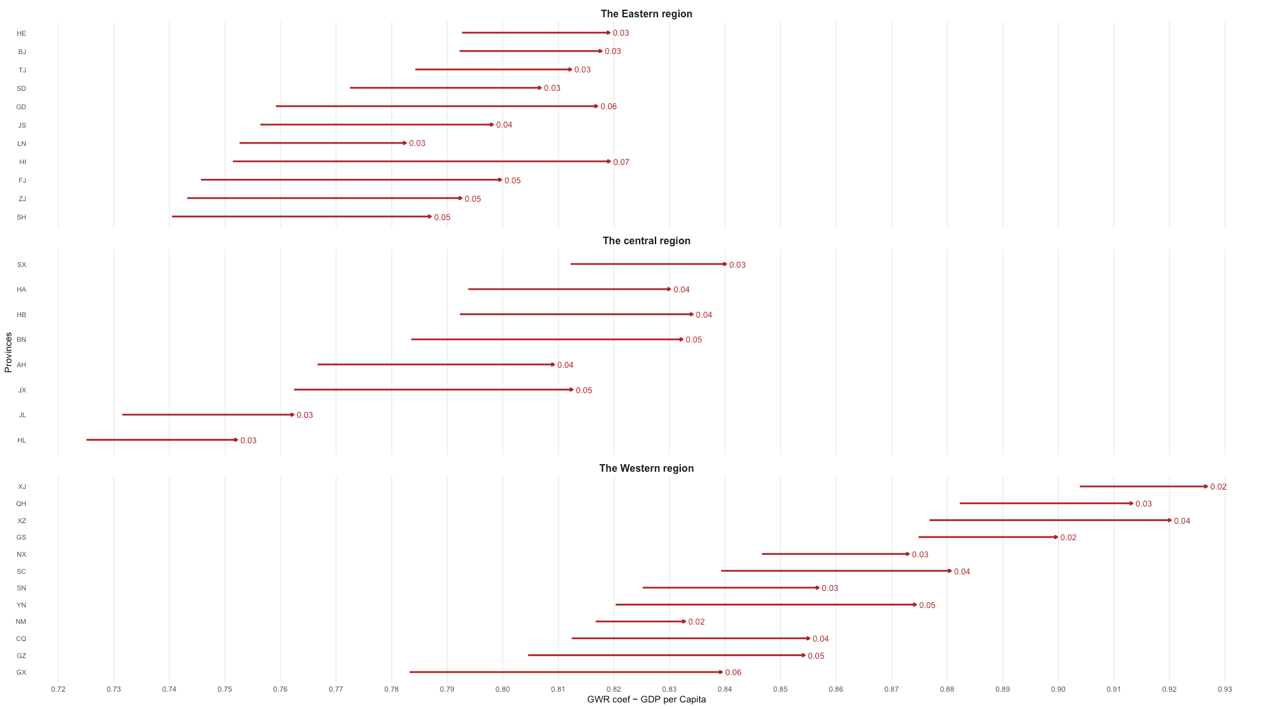


Supplementary Figure 5, Temporal changes of GWR result (Dependent variable, hospital-based availability; Independent variable, GDP per capita)


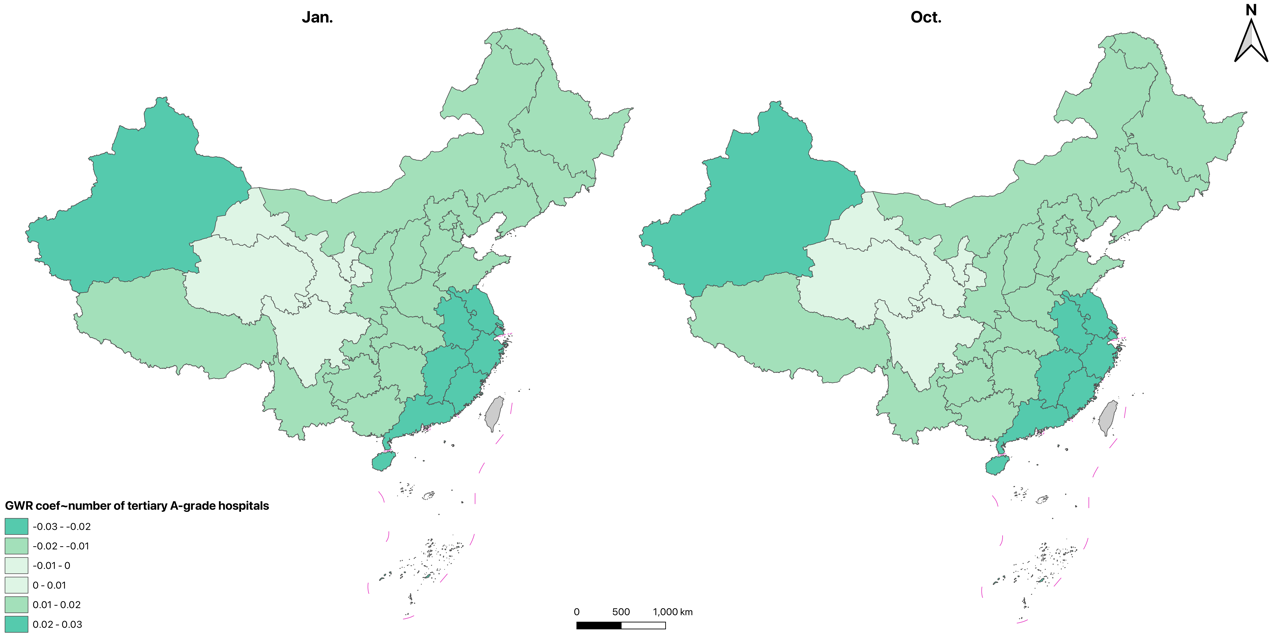


Supplementary Figure 6, GWR result (Dependent variable, hospital-based availability; Independent variable, number of tertiary A-grade hospitals)


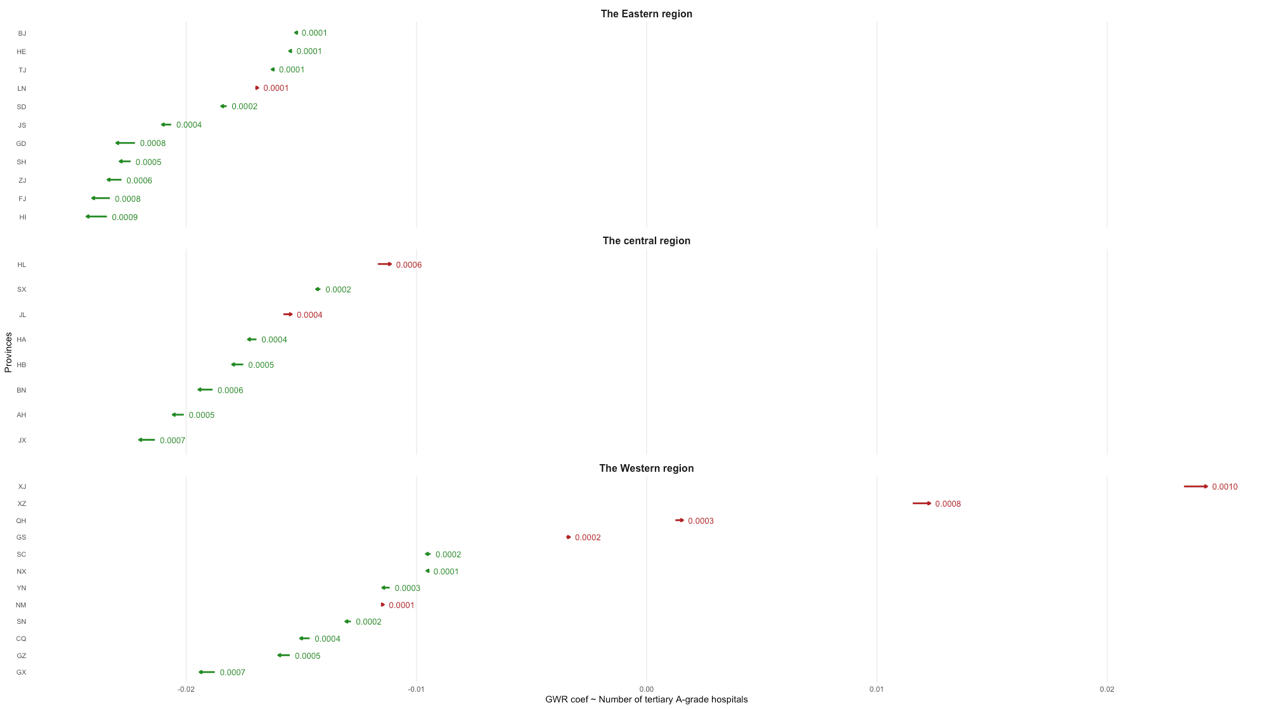


Supplementary Figure 7, Temporal changes of GWR result (Dependent variable, hospital-based availability; Independent variable, number of tertiary A-grade hospitals)


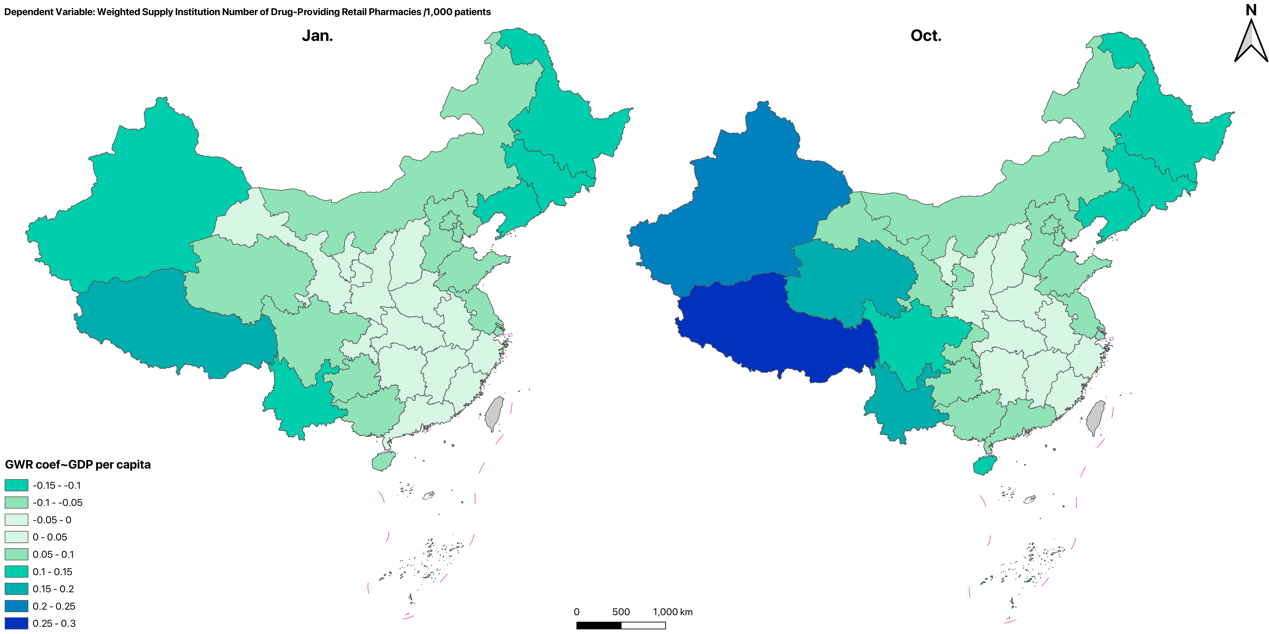


Supplementary Figure 8, GWR result (Dependent variable, retail pharmacy-based availability; Independent variable, GDP per capita)


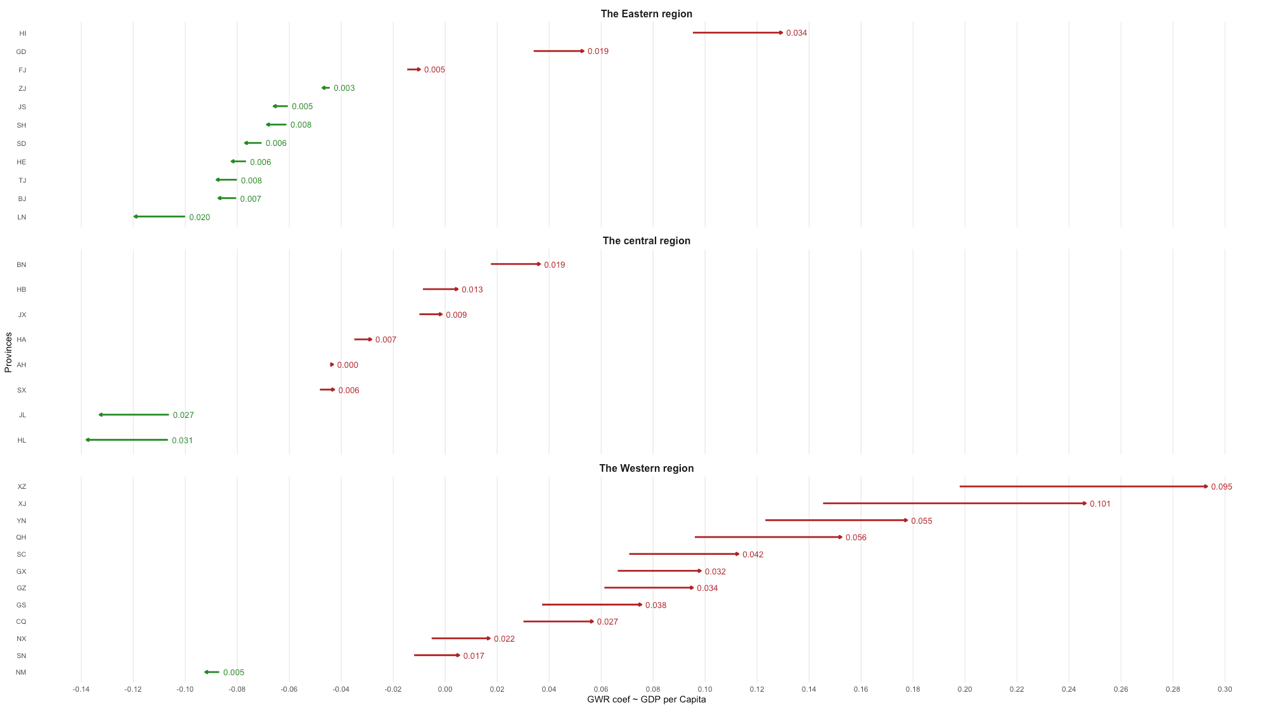


Supplementary Figure 9, Temporal changes of GWR result (Dependent variable, retail pharmacy -based availability; Independent variable, GDP per capita)


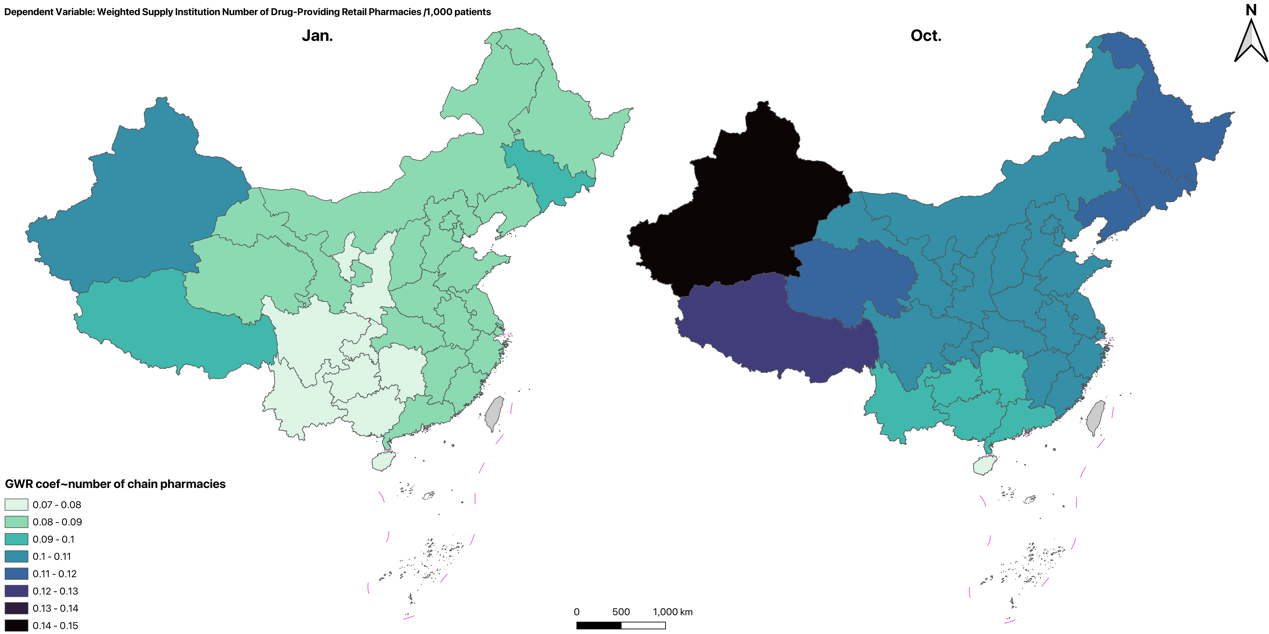


Supplementary Figure 10, GWR result (Dependent variable, retail pharmacy-based availability; Independent variable, number of chain pharmacies)


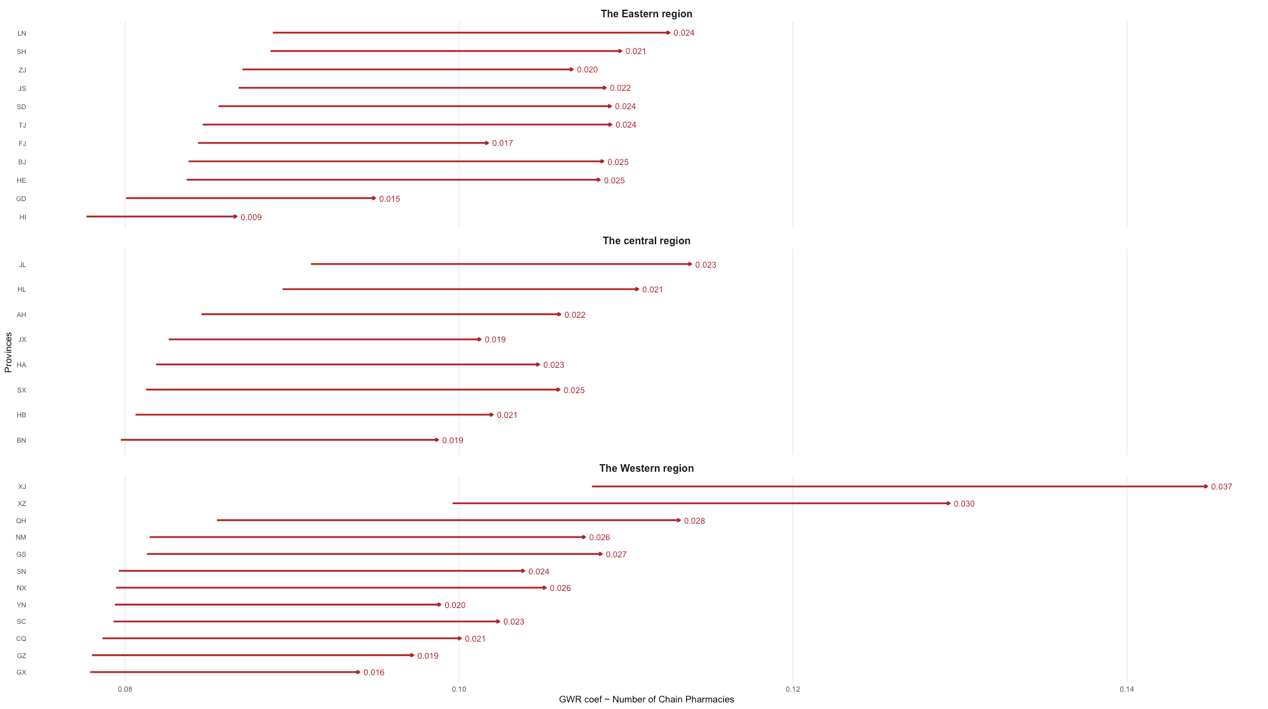


Supplementary Figure 11, Temporal changes of GWR result (Dependent variable, retail pharmacy-based availability; Independent variable, number of chain pharmacies)


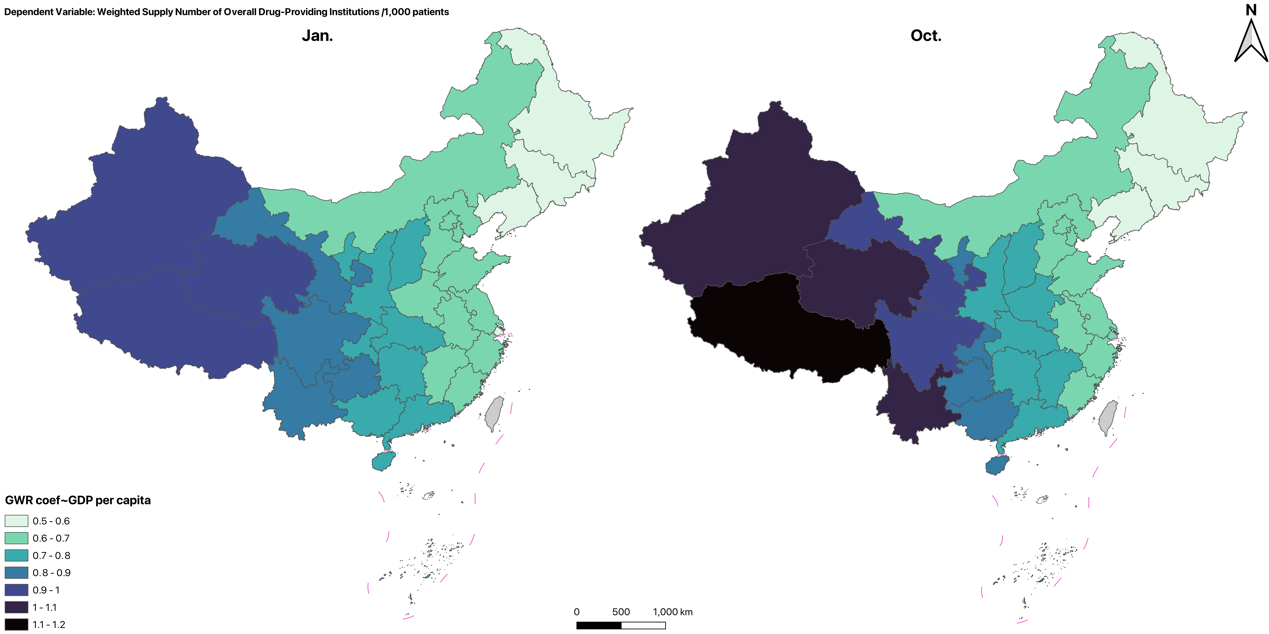


Supplementary Figure 12, GWR result (Dependent variable, overall availability; Independent variable, GDP per capita)


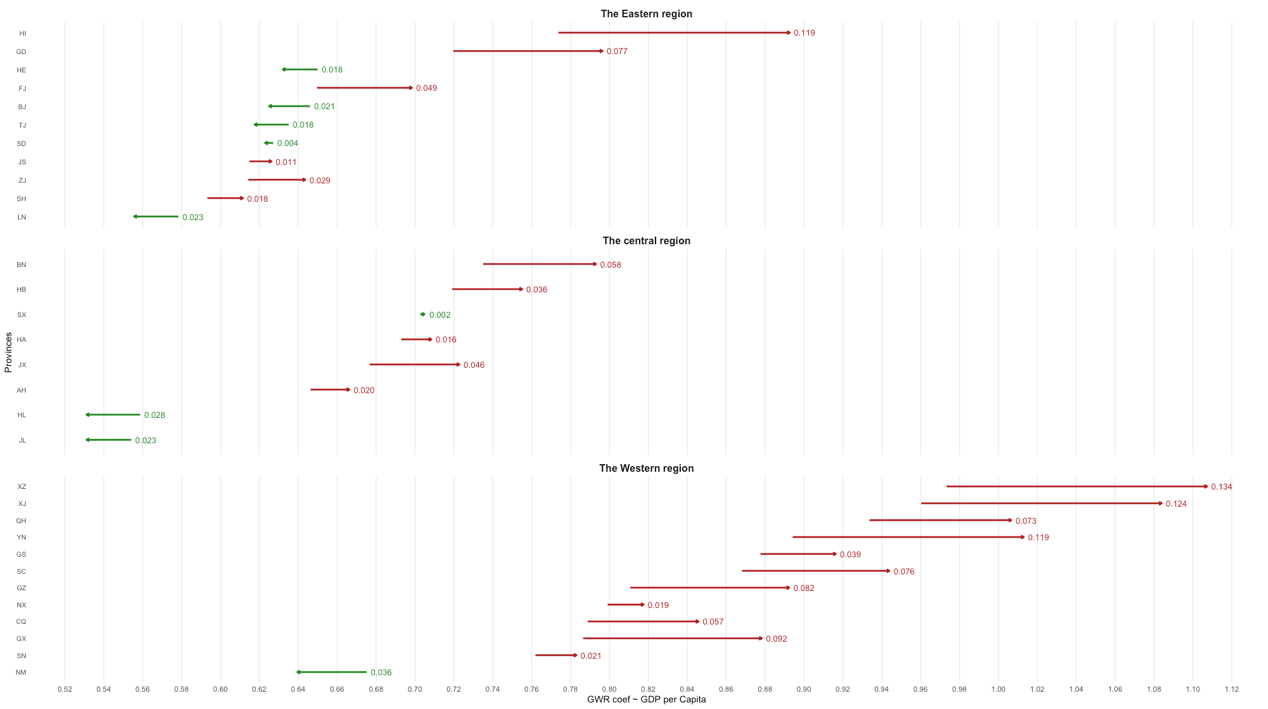


Supplementary Figure 13, Temporal changes of GWR result (Dependent variable, overall availability; Independent variable, GDP per capita)


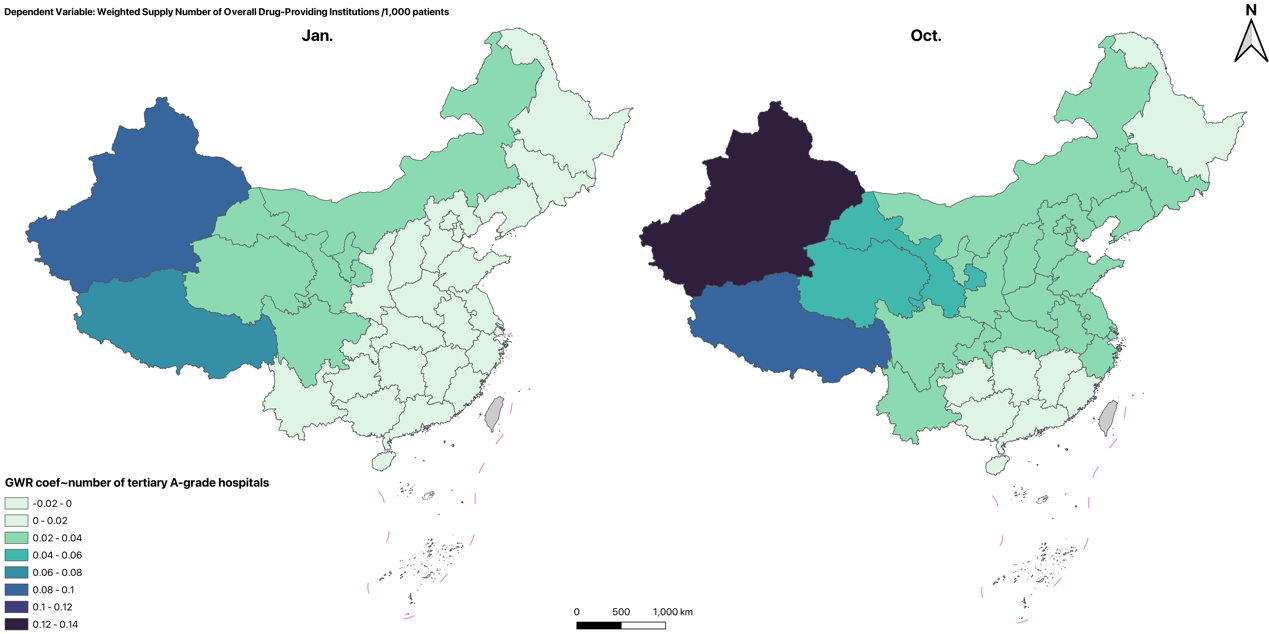


Supplementary Figure 14, GWR result (Dependent variable, overall availability; Independent variable, number of tertiary A-grade hospitals)


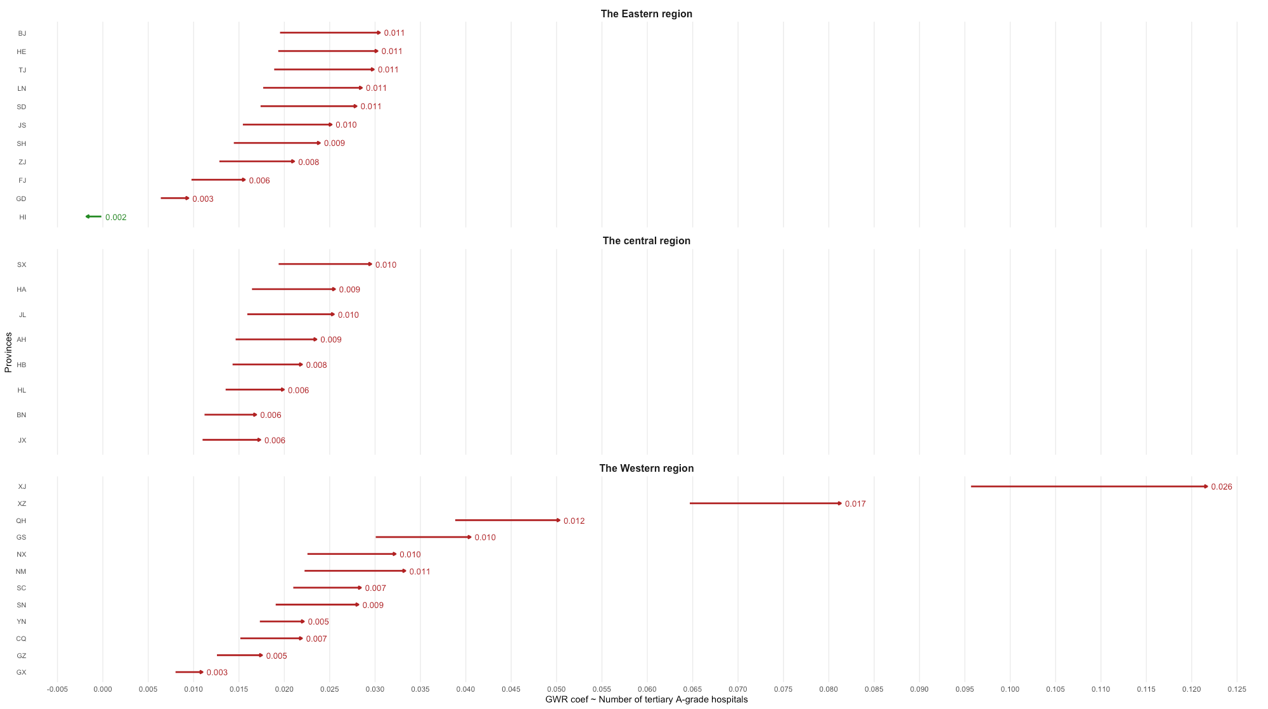


Supplementary Figure 15, Temporal changes of GWR result (Dependent variable, overall availability; Independent variable, number of tertiary A-grade hospitals)
